# Robust, high-density lesion mapping in the left atrium with near-infrared spectroscopy

**DOI:** 10.1117/1.JBO.29.2.028001

**Published:** 2024-02-28

**Authors:** Haiqiu Yang, Jonah A. Majumder, Ziyi Huang, Deepak Saluja, Kenneth Laurita, Andrew M. Rollins, Christine P. Hendon

**Affiliations:** a Columbia University, Department of Electrical Engineering, New York, United States; b Columbia University, Department of Biomedical Engineering, New York, United States; c Columbia University Irving Medical Center, Cardiology Division, Department of Medicine, New York, United States; d MetroHealth Hospital, Cardiology Division, Department of Medicine, Cleveland, Ohio, United States; e Case Western Reserve University, Department of Biomedical Engineering, Cleveland, Ohio, United States

**Keywords:** near-infrared spectroscopy, biomedical optics, cardiac ablation, cardiac electrophysiology

## Abstract

**Significance:**

Radiofrequency ablation (RFA) procedures for atrial fibrillation frequently fail to prevent recurrence, partially due to limitations in assessing extent of ablation. Optical spectroscopy shows promise in assessing RFA lesion formation but has not been validated in conditions resembling those *in vivo*.

**Aim:**

Catheter-based near-infrared spectroscopy (NIRS) was applied to porcine hearts to demonstrate that spectrally derived optical indices remain accurate in blood and at oblique incidence angles.

**Approach:**

Porcine left atria were ablated and mapped using a custom-fabricated NIRS catheter. Each atrium was mapped first in phosphate-buffered saline (PBS) then in porcine blood.

**Results:**

NIRS measurements showed little angle dependence up to 60 deg. A trained random forest model predicted lesions with a sensitivity of 81.7%, a specificity of 86.1%, and a receiver operating characteristic curve area of 0.921. Predicted lesion maps achieved a mean structural similarity index of 0.749 and a mean normalized inner product of 0.867 when comparing maps obtained in PBS and blood.

**Conclusions:**

Catheter-based NIRS can precisely detect RFA lesions on left atria submerged in blood. Optical parameters are reliable in blood and without perpendicular contact, confirming their ability to provide useful feedback during *in vivo* RFA procedures.

## 1 Introduction

Atrial fibrillation (AF) is a common cardiac arrhythmia associated with significant morbidity, mortality, and financial burden to the American healthcare system.^1^ Catheter-based radiofrequency ablation (RFA) is a minimally invasive AF treatment, in which radiofrequency electrical energy is used to electrically isolate presumed arrhythmogenic tissue.^2^ The aberrant electrical activity initiating AF has been shown to originate near the pulmonary veins (PVs) in a majority of patients, leading to widespread use of the procedure termed pulmonary vein isolation (PVI).^2^^,^^3^ However, arrhythmogenic tissue can exist beyond the PVs, limiting the effectiveness of PVI procedures.^4^^–^^6^ Also the success of RFA treatment is directly related to the depth and quality of RF energy delivery. Incomplete thermal treatment may permit subsequent conduction recovery, and many AF patients require a repeat procedure within 1 year of treatment.^5^ Conversely, tissue overtreatment can have serious complications, including cardiac perforation and embolic strokes.^7^ Hence, accurate characterization of ablated and unablated cardiac tissue is essential for the generation of safe, effective RF lesions that provide long-term freedom from atrial arrhythmias.

RFA is typically guided by electroanatomic mapping (EAM), a technique in which local electrograms are rendered into full 3D visualizations. These visualizations, along with catheter-based measurements of temperature, impedance, and power, guide clinicians in their placement of RFA lesions.^8^^–^^10^ However, the current EAM-based approach can lead to misinterpretation of ablation sites and cannot identify structural features.^11^ Various imaging modalities have been explored to better identify structural substrates and enhance monitoring and guidance of cardiac ablation therapy. Magnetic resonance imaging and computed tomography have both been explored for this purpose, but these approaches are costly and difficult to integrate with real-time feedback.^12^^–^^16^ Various ultrasound-based methods have also shown promise in monitoring cardiac ablation but are still in the development.^17^^,^^18^ Recently, optical imaging techniques have been applied to the challenge of RFA lesion characterization. Hyperspectral autofluorescence imaging has shown promise in localizing and characterizing RFA lesions, but the complexity of this technique (requiring a hyperspectral camera, UV illumination, and additional optics) renders it less compatible with endovascular access^19^^–^^21^ Optical coherence tomography has also proven capable of identifying diverse structural substrates, including fibrotic and adipose tissue,^22^^–^^27^ but tissue penetration depth is limited to 1 to 2 mm.

Near-infrared spectroscopy (NIRS) refers to the use of near-infrared light to characterize a specimen based on its wavelength-dependent optical properties of scattering and absorption. NIRS has recently been applied to the challenges of atrial substrate mapping and RFA lesion characterization.^28^^–^^30^ Human atrial wall thickness typically varies from 1 to 4 mm,^31^ roughly matching the penetration depth of near-infrared light into cardiac tissue.^30^ Prior studies from our group have demonstrated that catheter-based NIRS can reliably monitor RFA lesion depth and differentiate ablated from unablated tissue. However, these studies have all been performed under highly controlled conditions– in an optically transparent solution, with near-normal catheter incidence. This work aims to demonstrate that NIRS-based mapping is fast, insensitive to catheter angle, and unaffected by blood around the catheter tip. Because of these features, catheter-based NIRS is well-equipped to improve the safety and efficacy of RFA procedures.

## 2 Methods

### 2.1 Optical RFA Catheter and NIRS System

**Fig. 1:**
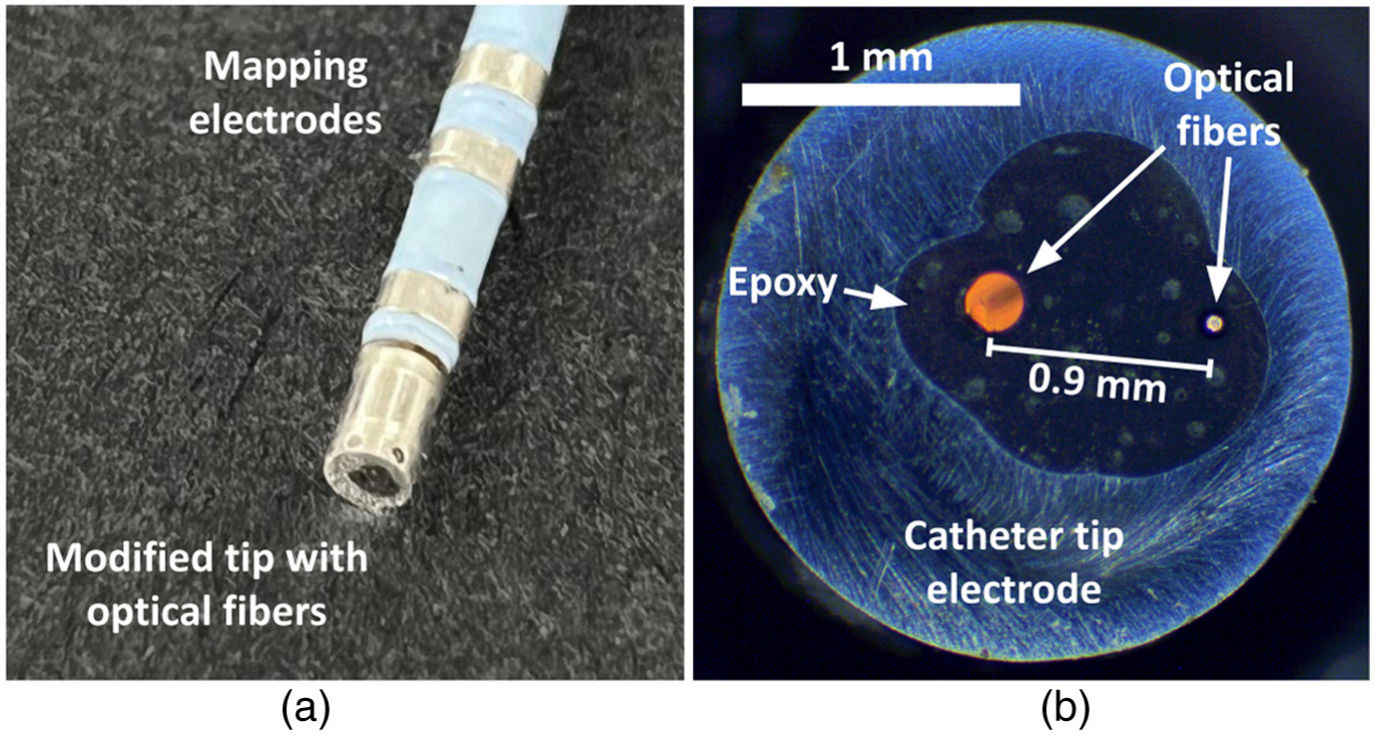
Commercial ablation catheter with integrated optical fibers for NIRS mapping. (a) Photograph of the catheter, showing the intact mapping electrodes as well as the modified tip. (b) Photomicrograph of the catheter tip illustrating the positions of the two multimode optical fibers.

A commercial open-tip irrigated Celsius thermocool RFA catheter (DI7TCDLRT, Biosense Webster, Irvine, California, United States) was modified to accommodate two multimode optical fibers, for illumination and detection. The illumination fiber (AFS50/125/145T, Fiberguide, Fairfax, Virginia, United States) had a core diameter of 50  μm and a full diameter (core and cladding) of 125  μm. The detection fiber (FG200LEA, Thorlabs, Newtown, New Jersey, United States) had a core diameter of 200  μm and a full diameter of 220  μm. The fiber tips were immobilized with epoxy during fabrication such that their separation distance was 0.9 mm, as shown in Fig. 1. This source–detector separation was chosen to maximize optical path length and sensitivity to chromophore absorption^32^ without interfering with existing internal hardware of the catheter, with outer diameter of 2.33 mm (7 French). The source fiber was connected to a broadband light source (HL-2000 HP, Ocean Optics Inc, Dunedin, Florida, United States) to illuminate the tissue surface. The emission spectrum of this light source ranged from 500 to 1100 nm, with nominal output power of 4.5 mW. The optical power measured at the catheter tip was 0.61 mW. Diffusely reflected light was collected by the detection fiber, subjected to a longpass filter with cutoff wavelength 600 nm (FELH0600, Thorlabs, Newton, New Jersey, United States), and analyzed by an NIR spectrometer (C9405CB, Hamamatsu, Bridgewater, New Jersey, United States) with a spectral range from 435 to 1145 nm and a resolution of 0.9 nm. The probe of an electromagnetic tracking system (TrakStar, Northern Digital Inc., Waterloo, Ontario, Canada) was mounted to the tip of the catheter in order to monitor the position and angle of the catheter. Our modifications do not compromise or interfere with the ability of the catheter to deliver RF ablation. Hence, used in conjunction with an RF generator (Stockert 70, Biosense Webster, Irvine, California, United States), the catheter is capable of delivering and continuously monitoring ablation (as we have previously demonstrated in Ref. 28). In addition, positional information from the tracking system allows for precise and instantaneous localization of the catheter tip for each acquired spectrum. Using this information, it is straightforward to construct visualizations of spectrally derived metrics with thousands of points and densities comparable to EAM.^10^ All experimental measurements were recorded and processed in MATLAB 2015b and 2019a (The Mathworks Inc., Natick, Massachusetts, United States).

### 2.2 Experimental Samples and RFA Protocol

**Fig. 2:**
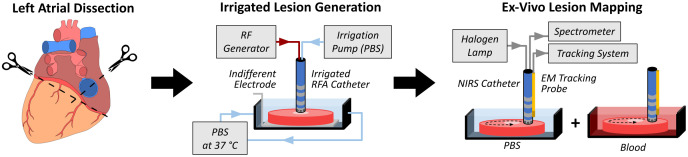
Experimental flow diagram of left atrial NIRS mapping. First, each left atrium was dissected away from the heart and flattened. Then irrigated lesions were generated in a line adjacent to a pulmonary vein. Finally, spectra were acquired from across the endocardium while the tracking system simultaneously recorded the catheter’s position.

Experiments were implemented on 22 healthy pig hearts (Green Village Packing Co., Green Village, New Jersey, United States). Each left atrium (including the atrial septum) was separated from ventricles and right atrium and sliced open to lie flat. The tissue was placed (with its endocardial surface up) on an electrosurgical grounding pad in a bath of phosphate buffered saline (PBS) at 37°C. First, irrigated lesions were created on the endocardial surface, with 25 to 45 W of RF power for 15 to 50 s, whereas an irrigation pump (CoolFlow, Biosense Webster, Irvine, California, United States) maintained a flow (of PBS) of 17  ml/min (based on clinical ablation settings) to the catheter tip. The variation in ablation parameters was intended to compensate for varying tissue thickness and avoid complications of overtreatment, such as steam-pops. Between 6 and 9 lesions were generated on each left atrium for a total of 173 lesions. Following lesion creation, left atria were placed in a separate chamber and pinned onto a corkboard for lesion characterization. For lesion characterization, all atria were mapped while submerged in both PBS or fresh porcine blood, to validate that NIRS-derived metrics retain sensitivity in blood. All imaging was completed within two days postmortem. The three main steps of this experimental protocol are depicted in Fig. 2.

Prior to mapping, a reference image of each dissected atrium was captured. For a handful of pixel locations in this reference image, the corresponding positions in physical space were recorded using the tracking system. These control points were used to fit a projective image transformation between physical space and image pixels, using MATLAB’s “fitgeotrans” command. Then the sample was submerged in PBS or blood, and NIR reflectance spectra were obtained for 500 to 2000 individual points, in a continuous acquisition, with a sampling rate of 2 Hz. At each point, the tracking system recorded the position (X, Y, and Z) and orientation (pitch, yaw, and roll) of the catheter tip. For each tracker-derived catheter position, the aforementioned image transformation was used to obtain the corresponding location (in pixels) on the reference image. In this way, the system provided real-time, image-registered visualization of the catheter tip location. The precise number of spectra obtained from each sample was chosen to fully cover the left atrial endocardium, excluding the left atrial appendage. Following NIRS mapping, atria were stained with 1% 2,3,5-triphenyl-2H-tetrazolium chloride (TTC) and further dissected to verify the extent of RFA lesions.

### 2.3 Spectral Processing and Optical Index Calculation

NIRS measurements were calibrated and absolute reflectance spectra were converted into relative reflectance spectra (denoted $R_{\mathrm{rel}}(\lambda )$) as follows. Raw spectra were first divided by reflectance from a diffuse (99%) reflectance standard (Spectralon, LabSphere, North Sutton, New Hampshire, United States) to compensate for the spectral shape of the light source and any other wavelength-dependent effects of the system.^33^ Next, spectra were scaled by the measured reflectance from a (polydimethylsiloxane) phantom at a wavelength with known optical properties.^34^^,^^35^ The resulting relative spectra were truncated to exclude wavelengths below 600 and above 1000 nm, lowpass filtered with cutoff frequency 0.05 π rad/sample, and renormalized to their values at 600 nm. This second, per-spectrum normalization was performed to address any remaining variation in spectrum amplitude not corrected by the aforementioned calibration scheme. The choice of normalization wavelength (600 nm) was mainly chosen for simple visualization and was also used in a previous publication.^28^ Derivative spectra are denoted $R_{\mathrm{rel}}^{\prime }(\lambda )$.

A previously published contact optical index (COI), calculated as shown in Eq. (1), was used to filter out points with poor catheter-tissue contact.^28^ The threshold value for the COI was evaluated based on the previously published mean COI values for contact and noncontact.^28^ Within this study, a threshold value of 0.93 was chosen, resulting in 18.5% of the spectra being rejected due to poor contact. After contact-based thresholding, 39,516 preprocessed spectra remained (22,821 from PBS and 16,695 from blood) across 22 atria. They were manually labeled (as either nonlesion or lesion) by comparing their recorded positions with the known positions of lesions. Then optical indices were computed for each acquired spectrum. Many of these optical indices have been previously demonstrated effective in differentiating ablated from unablated tissue, albeit in PBS, with perpendicular contact. The formulas for these optical indices are provided in Eqs. (2)–(5), (LOI, lesion optical index and SOI, substrate optical index). Note that the optical index denoted here as ${\mathrm{LOI}}_{0}$ was previously published as simply “LOI” and has been renamed here for clarity. One new ratiometric optical index [${\mathrm{SOI}}_{4}$, shown in Eq. (6)] was devised via a systematic search across all possible numerator–denominator combinations of integer wavelengths:

$$\mathrm{COI}=\frac{R_{\mathrm{rel}}(764\;\mathrm{nm})}{R_{\mathrm{rel}}(730\;\mathrm{nm})},$$ (1)

$${\mathrm{LOI}}_{0}=\overset{690\;\mathrm{nm}}{\underset{\lambda =660\;\mathrm{nm}}{\sum }}|R_{\mathrm{rel}}^{\prime }(\lambda )-R_{\mathrm{rel}}^{\prime }(952\;\mathrm{nm})|,$$ (2)

$${\mathrm{LOI}}_{8}=R_{\mathrm{rel}}^{\prime }(601\;\mathrm{nm})-R_{\mathrm{rel}}^{\prime }(609\;\mathrm{nm}),$$ (3)

$${\mathrm{SOI}}_{1}=\overset{1000\;\mathrm{nm}}{\underset{\lambda =600\;\mathrm{nm}}{\sum }}|R_{\mathrm{rel}}^{\prime }(\lambda )-R_{\mathrm{rel}}^{\prime }(661\;\mathrm{nm})|,$$ (4)

$${\mathrm{SOI}}_{3}=\frac{R_{\mathrm{rel}}(650\;\mathrm{nm})}{R_{\mathrm{rel}}(930\;\mathrm{nm})},$$ (5)

$${\mathrm{SOI}}_{4}=\frac{R_{\mathrm{rel}}(630\;\mathrm{nm})}{R_{\mathrm{rel}}(600\;\mathrm{nm})}.$$ (6)

**Fig. 3:**
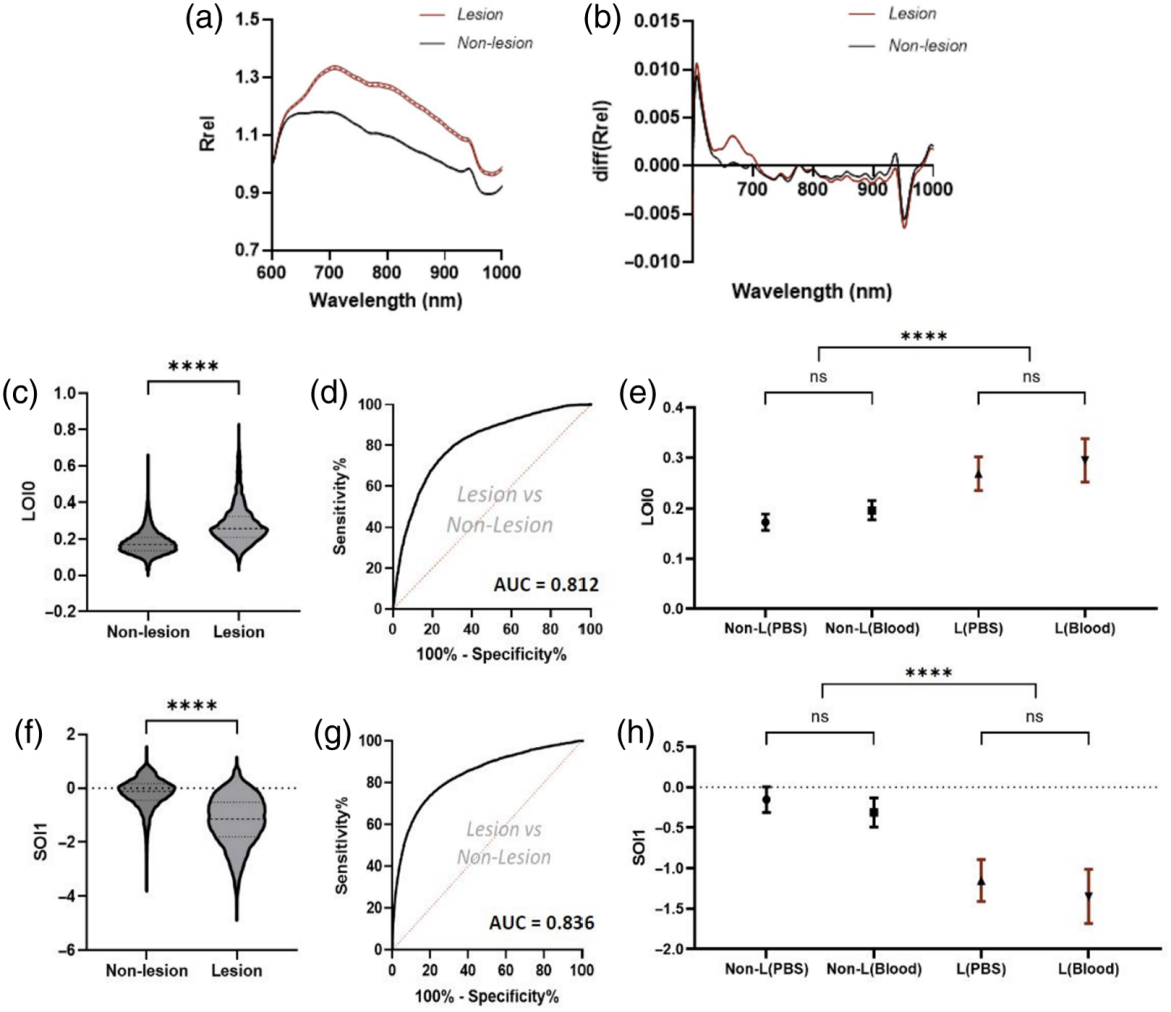
Spectral morphologies and optical index analysis for two key optical indices. (a) Mean (dotted line) and 95% confidence interval (shaded region) relative reflectance spectra for nonlesion and lesion tissue. (b) Relative reflectance first-order derivative spectra for nonlesion and lesion tissue. (c) Distributions and t test results for ${\mathrm{LOI}}_{0}$ between nonlesion and lesion tissue. (d) Hypothetical ROC curve and AUC, using ${\mathrm{LOI}}_{0}$ as an independent classifier. (e) Mean per-heart ${\mathrm{LOI}}_{0}$ values (with 95% confidence intervals) for nonlesion and lesion tissue, in PBS and blood; (f) Distributions and t test results for ${\mathrm{SOI}}_{1}$ between nonlesion and lesion tissue. (g) Hypothetical ROC curve and AUC, using ${\mathrm{SOI}}_{1}$ as an independent classifier. (h) Mean per-heart ${\mathrm{SOI}}_{1}$ values (with 95% confidence intervals) for nonlesion and lesion tissue, in PBS and blood (****p<0.0001, ns: p>0.05).

For each optical index, a two-tailed t-test was performed against the null hypothesis of equal means for nonlesion and lesion tissue (pooling data across all hearts). Receiver operating characteristic (ROC) curves were generated and the corresponding areas under the curve (AUCs) were computed to assess the classifying capability of each optical index. Next, for each of the 44 maps (22 in PBS and 22 in blood), a per-heart mean optical index value was calculated for both classes (i.e., nonlesion and lesion). This yielded, for each optical index, 88 values: 22 for nonlesion tissue in PBS, 22 for lesion tissue in PBS, 22 for nonlesion tissue in blood, and 22 for lesion tissue in blood. Two-sided t-tests were performed to compare the mean values between PBS and blood for nonlesion and lesion. Figure 3 illustrates this statistical analysis for two optical indices, ${\mathrm{LOI}}_{0}$ and ${\mathrm{SOI}}_{1}$. All signal processing and statistical analysis were performed in MATLAB 2019a and GraphPad Prism 9.4.1 (GraphPad Software, United States).

**Fig. 4:**
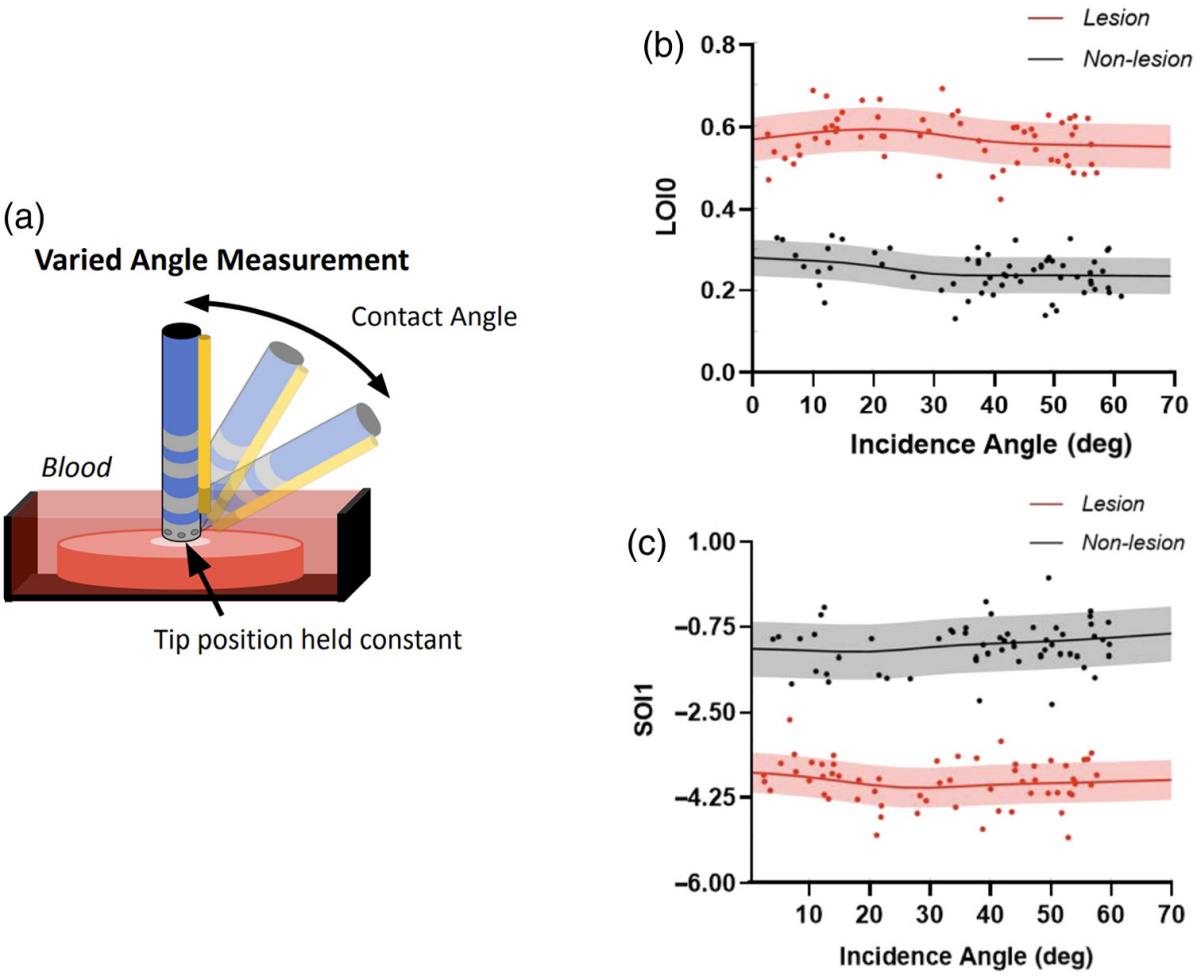
Contact angle sensitivity assessment. (a) Depiction of dedicated experiments conducted to acquire data at varying catheter contact angles, from a single location. (b) ${\mathrm{LOI}}_{0}$ data acquired at varying contact angles on lesion and nonlesion tissue. (c) ${\mathrm{SOI}}_{1}$ data acquired at varying contact angles on lesion and nonlesion tissue. In panels (b) and (c), lines and shaded regions illustrate sliding kernel-weighted estimates of mean and standard deviation.

A separate, dedicated experiment on two left atria was conducted to evaluate the sensitivity of NIRS measurements to catheter incidence angle. With the location of the catheter tip held constant, the angle of the catheter was systematically varied from normal incidence (0 deg) to ∼70  deg, as illustrated by Fig. 4(a). Afterward, COI-based thresholding (as described above) was used to exclude spectra obtained with poor contact. This analysis was conducted separately on untreated and ablated left atrial tissue, with samples submerged in blood. Spectra were processed into optical indices as detailed above. The relationship between contact angle and each optical index value could then be evaluated for nonlesion and lesion tissue.

### 2.4 Classification Algorithm

The classification dataset was constructed by calculating a feature vector of optical indices from each individual spectrum,^28^ including both PBS and blood data. Correlations between features were assessed using the Pearson correlation coefficient, and five representative uncorrelated features were selected to be used in the classification model for the lesion and nonlesion tissue. The dataset was divided into training (32,392 spectra from 18 hearts) and testing (7124 spectra from 4 hearts) groups. The training set contained 28,482 spectra (87.9%) labeled as nonlesion and 3910 (12.1%) labeled as lesion, and the testing set contained 6061 (85.1%) labeled as nonlesion and 1063 (14.9%) labeled as lesion. Five different machine learning classification algorithms were trained and their results compared on the basis of ROC AUC. These models were logistic regression, k-nearest neighbors (kNN), extreme gradient boosting (XGB), a support vector machine (SVM), and a random forest (RF). For each model, five-fold cross validation was used within the training set, and the final trained model parameters were obtained by averaging the five results. Class weights were tuned to alleviate the impact of class imbalance. Model training and testing were implemented in MATLAB and RStudio (Posit, Boston, Massachusetts, United States).

### 2.5 Blood Mapping Robustness

To assess the equivalence of mapping in PBS and blood, an additional analysis was performed to directly compare model-predicted lesion probabilities for hearts within the test set. First, in order to apply traditional image-processing techniques, scattered points were (linearly) interpolated onto a uniform grid. This interpolation was applied to both the model-predicted probabilities and the ground truth labels. The scattered points from both PBS and blood data were used to define a common concave boundary, outside of which, interpolated data were excluded from analysis. Interpolated data were subjected to a 2D lowpass filter with cutoff frequency 0.1 π rad/sample.

Interpolated model probability data were compared using three different image comparison metrics: structural similarity (SSIM) index,^36^ Frobenius normalized inner product, and root-mean-squared (RMS) difference. Only points within the predefined boundary were included in these computations. Maps were compared after thresholding probabilities into binary lesion/nonlesion predictions. Binary prediction maps in PBS and blood were compared using the Dice coefficient.

## 3 Results

### 3.1 Optical Index Assessment

#### 3.1.1 Statistical analysis

Analysis of spectral morphology and individual optical index values showed clear differences between nonlesion and lesion tissue. Mean spectra and mean derivative spectra, for lesion and nonlesion points, are shown in Figs. 3(a) and 3(b), respectively. These show that (after normalization by reflectance at 600 nm), lesion tissue exhibits greater reflectance from 700 to 900 nm and a larger derivative from 650 to 700 nm.

${\mathrm{LOI}}_{0}$ and ${\mathrm{SOI}}_{1}$ were two optical indices exhibiting statistical significance between mean values for lesion and nonlesion points [Figs. 3(c) and 3(f)]. Using these optical indices alone as binary classifiers, the ROC AUCs were 0.812 and 0.836, respectively, [Figs. 3(d) and 3(g)]. Comparing per-heart optical index values, the mean ${\mathrm{LOI}}_{0}$ (n=44 maps) and ${\mathrm{SOI}}_{1}$ (n=44 maps) values did not exhibit significant differences between PBS and blood, for both lesion and nonlesion tissue [as shown in Figs. 3(e) and 3(h)].

#### 3.1.2 Angle sensitivity analysis

The results of the independent angle analysis suggested broad insensitivity to contact angle for all optical indices included as model features. After contact thresholding, the contact angles ranged from 0 deg to ∼60  deg. Shown in Figs. 4(b) and 4(c) are the measured optical index values for this range of contact angles, for ${\mathrm{LOI}}_{0}$ and ${\mathrm{SOI}}_{1}$. At contact angles up to 60 deg, optical index values for nonlesion and lesion tissue remained distinct. Similar results were obtained for other included optical indices.

### 3.2 Classification Results

**Fig. 5:**
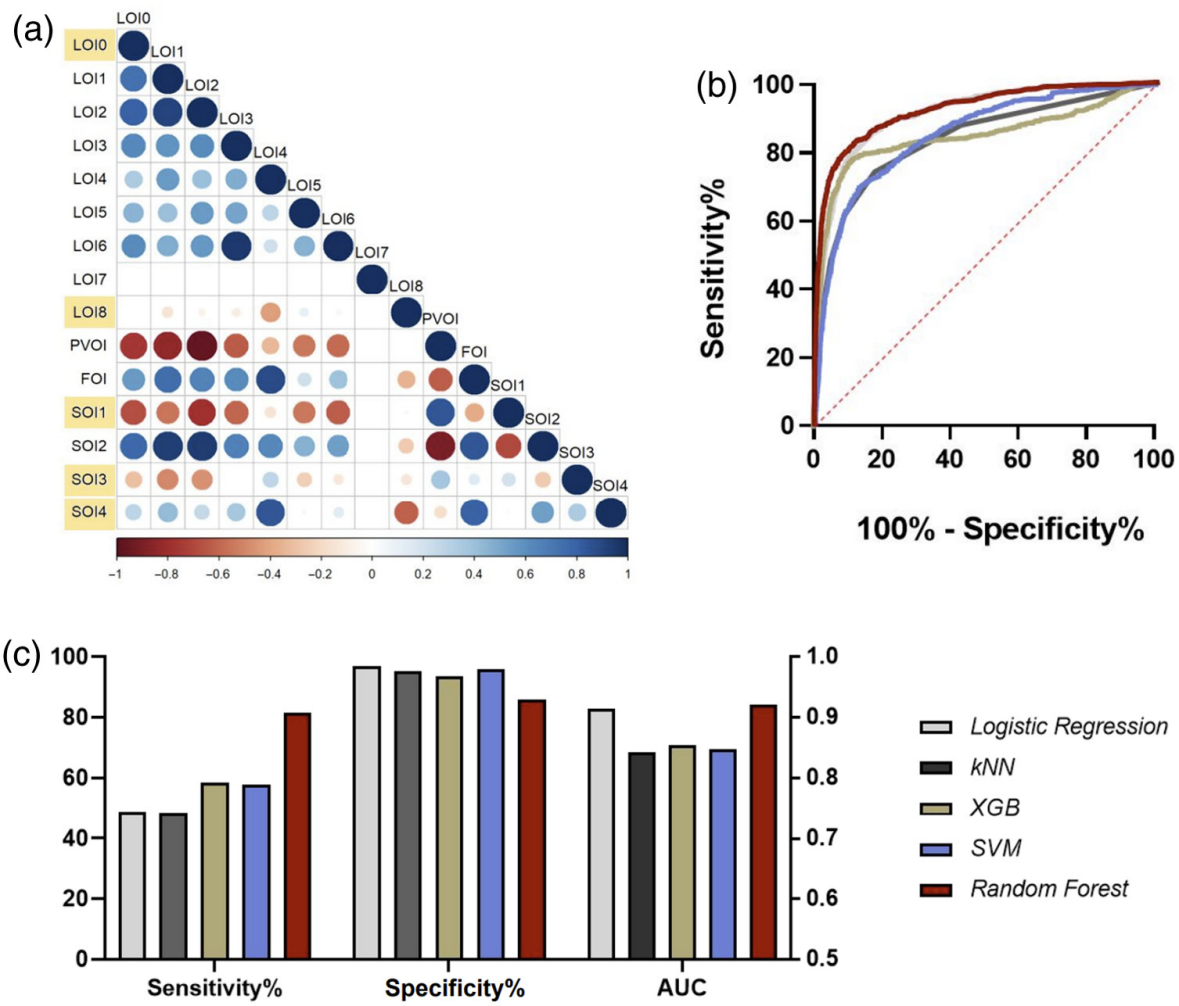
Assessment of machine learning classifiers. (a) Candidate feature correlation matrix with selected features (i.e., optical indices) highlighted in yellow. (b) ROC curves for tested classifiers. (c) Sensitivity, specificity, and ROC AUC values for tested models (with a probability threshold of 0.5).

Fifteen optical indices were considered as potential features in the classification model [listed in Fig. 5(a)]. ${\mathrm{LOI}}_{0}$ and ${\mathrm{SOI}}_{1}$ were preserved for the final model, due to statistical performance illustrated above and in a previous study.^28^ Three other optical indices were chosen for inclusion based on low correlations with ${\mathrm{LOI}}_{0}$, ${\mathrm{SOI}}_{1}$, and each other [highlighted in Fig. 5(a)]. These optical indices were ${\mathrm{LOI}}_{8}$, ${\mathrm{SOI}}_{3}$, and ${\mathrm{SOI}}_{4}$ with individual AUC values ranging from 0.517 to 0.643.

All five machine learning models were evaluated on the test set, generating the ROC curves and AUCs shown in Fig. 5(b). All models were effective in the classification of lesion and nonlesion spectra, validating the choice of input features. When thresholded at a probability value of 0.5, all models exhibited high specificity, and the RF classifier yielded the highest sensitivity (81.66%, compared with 48.64%, 48.26%, 57.42%, and 57.86%, for logistic regression, kNN, XGB, and SVM, respectively). The RF model also led to a higher AUC (0.921) than the other tested models [0.914, 0.842, 0.854, and 0.848, shown in Fig. 5(c)].

**Fig. 6:**
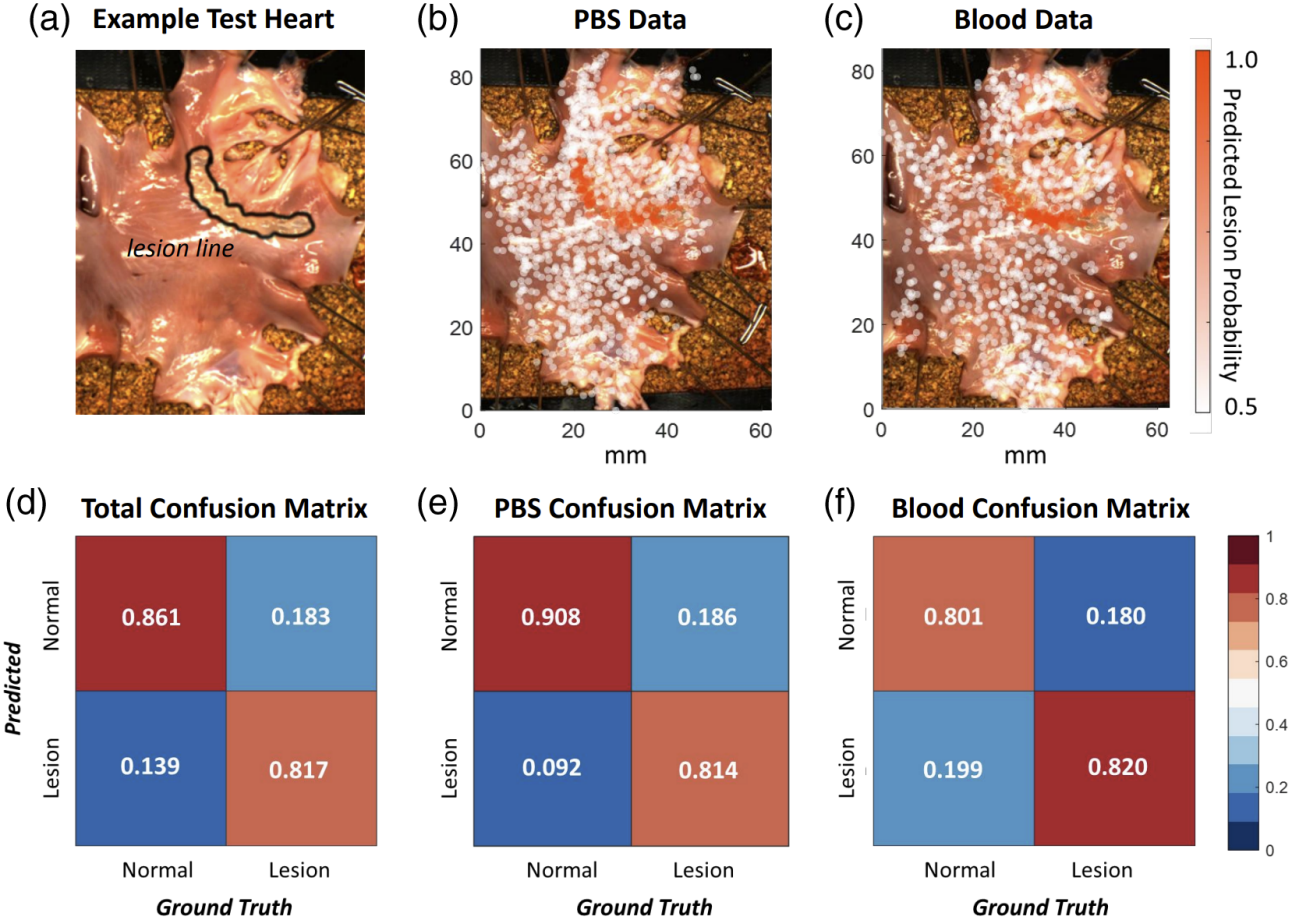
Lesion classification results for an example heart in the test set. (a) Reference image showing the known locations of a line of 9 lesions generated near a pulmonary vein. (b) Predicted lesion probabilities from data collected with the heart submerged in PBS. (c) Predicted lesion probabilities from data collected with the heart submerged in blood. (d) Confusion matrix output for the trained RF model applied to the full test set, after applying a probability threshold of 0.5. (e) Confusion matrix output for (test set) PBS data. (f) Confusion matrix output for (test set) blood data. In panels (b) and (c), each point represents a single acquired spectrum.

Based on these results, the trained RF classifier was selected as the final model. An example comparing model predictions to ground truth data for test heart 1 is shown in Figs. 6(a)–6(c). Figure 6(a) shows the reference image with the true lesion locations outlined (intentionally generated alongside the boundary of one flattened PV). Figures 6(b) and 6(c) show (on the same reference image) the tracker-recorded catheter positions of acquired spectra, colored by model-predicted lesion probability. Data in Fig. 6(b) were acquired in PBS, whereas data in Fig. 6(c) were acquired in blood. Predicted lesion probabilities match ground truth locations well, regardless of mapping environment. A probability threshold of 0.5 yielded the overall confusion matrix shown in Fig. 6(d), when applied to the entire test dataset. Applying this threshold individually to the PBS and blood test data yielded the confusion matrices in Figs. 6(e) and 6(f).

### 3.3 Blood Mapping Robustness

**Fig. 7:**
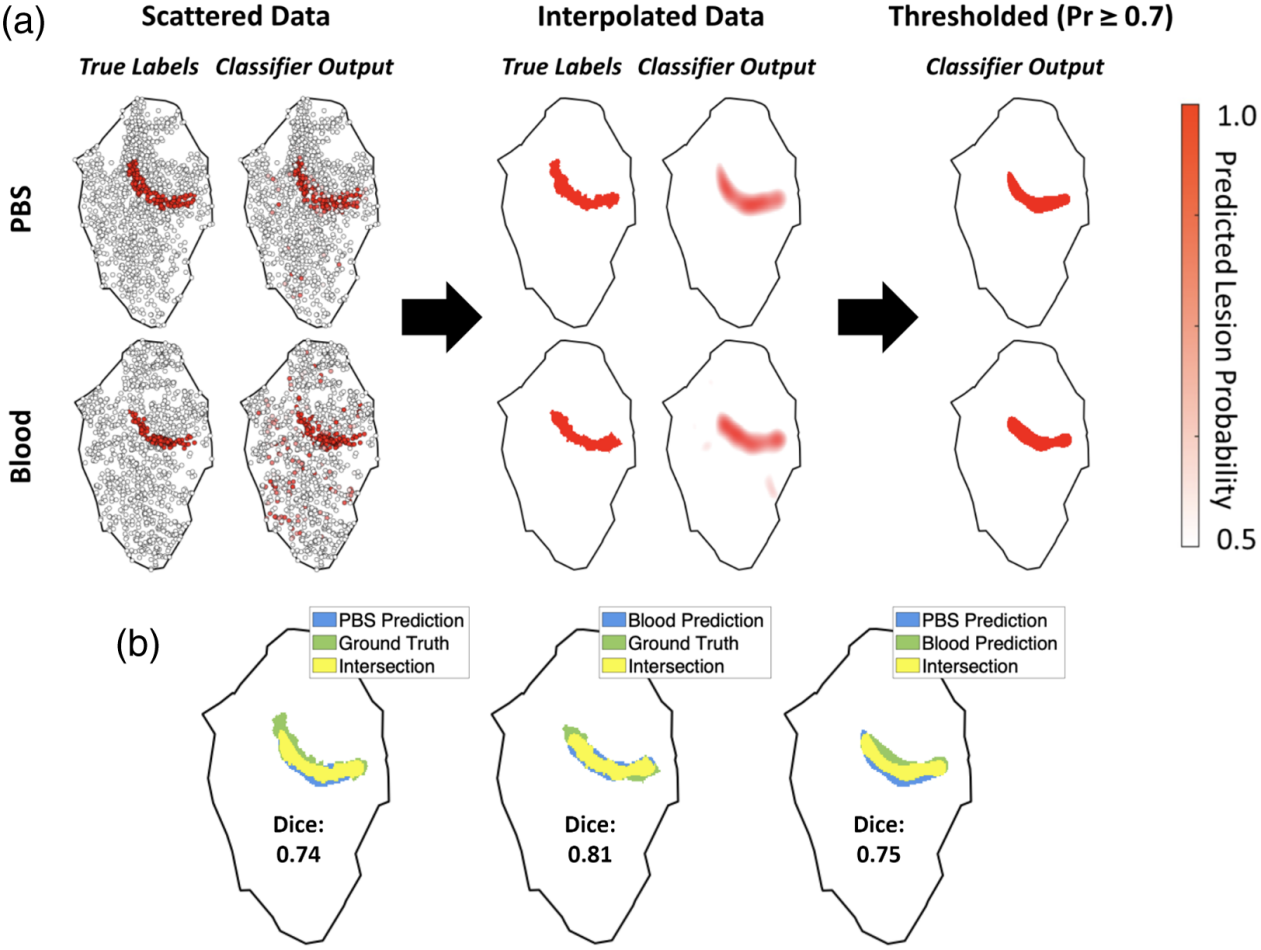
Example comparison between data collected from test heart 1, submersed in both PBS and blood. (a) From left to right: scattered model output (lesion probabilities); model output after interpolation onto a uniform grid; model output after thresholding with a probability value of 0.7. Scattered, grid-interpolated, and thresholded data are all visualized with a common color scale (a probability range of 0.5 to 1.0). (b) From left to right: thresholded predictions from PBS-mapped data compared with ground truth; thresholded predictions from blood-mapped data compared with ground truth; thresholded predictions from PBS- and blood-mapped data compared with one another.

Data from all four test hearts were processed and individually compared as described in Sec. 2.5. The procedure for obtaining thresholded binary predictions and computing Dice coefficients is illustrated (for test heart 1) in Fig. 7. Calculated image comparison metrics are reported in Table 1. Across all test hearts, the mean SSIM value was 0.749, the mean inner product value was 0.867, and the mean RMS difference was 0.186. The mean Dice coefficients, for threshold values of 0.3, 0.5, and 0.7, were 0.571, 0.653, and 0.421. The Dice coefficient for test heart 3 with a threshold value of 0.7 is not listed because both the PBS- and blood-based predictions had no points with predicted probabilities above 0.7. The degree of similarity between maps obtained in PBS and blood, quantified by these metrics, suggests that NIRS measurements are equivalent in the two media.

**Table 1:** Results of direct comparisons between PBS- and blood-mapped test set hearts. The example heart shown throughout Figs. 6 and 7 is labeled as test heart 1. The dashes in the rightmost column for test heart 3 signify that at a probability threshold of 0.7, the model predicted no lesions for either the PBS or blood map.

	Continuous probabilities			Thresholded predictions		
Test heart	SSIM	Inner Prod.	RMS Diff.	Dice (P≥0.3)	Dice (P≥0.5)	Dice (P≥0.7)
1	0.726	0.901	0.149	0.476	0.870	0.747
2	0.741	0.846	0.158	0.612	0.572	0.363
3	0.769	0.868	0.138	0.600	0.582	—
4	0.760	0.853	0.300	0.596	0.587	0.572

## 4 Discussion

In this work, we have demonstrated high-density lesion mapping with an NIRS-integrated RFA catheter on *ex vivo* porcine left atria. The use of an electromagnetic tracking system facilitated faster sampling, real-time display of image-registered spectra, and dense tissue mapping. These features stand in contrast with the previously published camera tracking-based NIRS system that was capable of acquiring only a few hundred spectra within 30 min and required visualizations to be generated afterwards, offline.^29^ Optical measurements were validated with respect to reliability in blood and insensitivity to contact angle, and an RF classifier was proposed to predict lesion probability. Predicted lesion spatial distributions were visualized as 2D maps according to positions recorded by the tracking system. Additionally, the equivalence of mapping in PBS and blood was assessed through multiple comparison metrics (SSIM, inner product, RMS difference, and Dice coefficient).

All tested machine learning models performed reasonably well (all AUCs>0.8) distinguishing nonlesion from lesion tissue in test set hearts (shown in Fig. 5). This confirms that the chosen optical indices capture the clear inherent differences in the spectral signatures of ablated and unablated tissue. Prior studies have shown that ablation of myocardial tissue both increases scattering and changes absorption through the creation of metmyoglobin.^37^^,^^38^ Still, the optical indices used here are not the only possible approach to spectral analysis, and dimensionality reduction techniques, such as principal component analysis might prove helpful to identify important wavelengths in future studies.

The RF model achieved the highest classification accuracy within the test set, both when data were pooled [Fig. 6(d)] and assessed individually in PBS and blood [Figs. 6(e) and 6(f)]. The fact that the raw output takes the form of a (lesion) probability is an advantage of this model. When a binary decision is desired, this affords the user the flexibility to tailor the cutoff probability to the application. During RFA procedures, when the risk of electrical reconnection is high, a low threshold can be used to prioritize sensitivity over specificity. Conversely, a high threshold can be used to prioritize specificity and avoid overtreatment.

Compatibility with large contact angles is crucial for thorough *in vivo* left atrial mapping because the 3D geometry of the atria often prevents ideal, perpendicular contact angles. The effects of poor contact can easily confound the effects of oblique contact angles: at large angles, it becomes more difficult to ensure good catheter-tissue contact.^9^^,^^28^ However, our data suggest that (as long as contact is maintained) changes in contact angle to around 60 deg do not compromise the tissue-discriminating ability of NIRS. This is consistent with physical models of diffuse reflectance spectroscopy, which suggests that while catheter angle can affect the sampled volume, the system remains sensitive to tissue optical properties.^34^

Blood presents a challenge for all intracardiac and intravascular optical imaging technologies since it is not transparent and has a significant absorption spectrum.^39^^,^^40^ The success of our classification algorithm [particularly for blood-only data, shown in Fig. 6(f)] suggests that surrounding blood does not affect the reliability of NIRS-derived measurements. Quantitative comparison metrics between PBS- and blood-mapped test hearts (all SSIM values >0.7 and all inner products >0.8) also support this idea. It should be noted that these results were derived from the subset of data where good catheter-tissue contact was maintained, and attempting to classify spectra with poor or no contact would likely yield worse results. However, this is not a limitation of our system because contact-based thresholding allows for identification and exclusion of points with poor contact. Ultimately, these results indicate that our catheter is capable of mapping lesions in blood whenever it is in contact with the tissue.

Previous studies suggest that NIRS can detect RFA lesion morphology and depth.^28^ In our data, some lesions contained more tissue damage than others, owing to complicated interactions between ablation parameters, tissue thickness, and underlying tissue composition. This is a potential reason for varying predictions across different lesions and hearts. Similarly, the edge of lesion is not a well-demarcated boundary but a gradual transition from the maximally ablated central region to the surrounding untreated tissue.^35^ This may have led to inaccurate classification at lesion gaps and boundaries.

Future work will aim to address limitations of this study. First, pulmonary venous tissue was excluded from the analysis because it was frequently too thin to acquire spectra unaffected by the underlying corkboard. Modification of the dissection procedure and tissue mounting strategy could mitigate this problem. While a temperature of 37°C was maintained for ablation, mapping was performed in baths at room temperature (for logistical reasons). Bath temperature is not expected to affect optical measurements, but this should be verified. Next, healthy pig atria approximate human atria well in modeling the effects of RFA-induced changes, but they do not exhibit the pathologic changes that have been implicated in AF initiation and maintenance.^41^^–^^44^ In the future, NIRS data will be collected from diseased human hearts in order to incorporate these pathologic features into the classification algorithm. Finally, while efforts were made to replicate the environment of a genuine RFA procedure, these results will need to be validated with *in vivo* large animal experiments.

## Data Availability

The data presented in this article are publicly available in Columbia University Academic Commons at https://doi.org/10.7916/nhej-0s11.
